# The Epidemiological Relevance of Family Study in Chagas Disease

**DOI:** 10.1371/journal.pntd.0001959

**Published:** 2013-02-14

**Authors:** Inés Zulantay, Werner Apt, Daniel Ramos, Lorena Godoy, Claudio Valencia, Matías Molina, Eduardo Sepúlveda, Patricio Thieme, Gabriela Martínez, Gabriela Corral

**Affiliations:** 1 Laboratory of Basic Clinical Parasitology, Program of Cellular and Molecular Biology, Institute of Biomedical Sciences, Faculty of Medicine, University of Chile, Santiago, Chile; 2 Salamanca Hospital, Coquimbo Health Service, IV Region, Chile; 3 Combarbalá Hospital, Coquimbo Health Service, IV Region, Chile; 4 Illapel Hospital, Coquimbo Health Service, IV Region, Chile; René Rachou Research Center, Brazil

Chagas disease is a public health problem in Latin America [1], and for the last 12 years or so has been an emergent epidemiological situation in countries with immigrants from endemic areas [2], [3]. Although there have been important advances in the vector and transfusion control of this parasitosis for endemic areas with a history of domestic infestation [4], there is little information that would allow an estimation of the number of family members infected with *Trypanosoma cruzi*.

**Figure 1:**
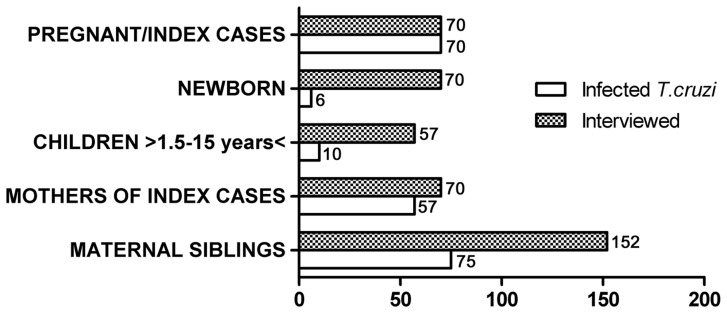
Infection by *Trypanosoma cruzi* in the maternal line of 70 family groups considering as index cases women with Chagas disease confirmed serologically by ELISA and IFI IgG during pregnancy. Total members interviewed: 419. Total members with Chagas disease: 218 (mean per familiy: 3.1).

In order to provide this information, between the years 2008 and 2011 we studied infection by *T. cruzi* in maternal lines considering as index cases 70 women with chronic Chagas disease, confirmed serologically during the first third of their pregnancies with ELISA (Chagas III kit, GrupoBios SA, Chile) and indirect immunofluorescence (IFI) IgG (in-house). Of the 504 members of the family groups of the 70 index cases, 419 persons who were surveyed (coverage 83.1%) agreed to participate in this study under written informed consent approved by the Ethics Committee of the Faculty of Medicine of the University of Chile (Figure 1) (Resolution 1613/December 2007).

All the index cases came from rural (62.9%) and urban (37.1%) sectors of the Province of Choapa, IV Region, Chile (an endemic area whose vector, *Triatoma infestans*, has been controlled) [5]. The ages of the women ranged from 18 to 47, with a mean of 31.3 years. The total number of pregnancies in the index group was 159 (range 1–5); they had 144 live children. Ninety-one percent of the women did not work outside the home and 75.7% had finished grammar school (8 years), but only 30% had finished high school. These women know the vector; 88.6% said they recognize the insect, 81.4% clearly remember its presence in the houses of their childhood, and 40 of them (57%) mentioned having been bitten by them. During their childhood, 91.4% of the women lived in a house with sun-baked bricks and/or a thatched roof. Currently, 84.2% live in improved or solid houses, which reflect the government housing policy of the last few decades [6]. Educational interviews with the index cases during their pregnancy revealed fears, myths, and lack of information about basic aspects of Chagas disease. In part due to the education acquired, all mothers authorized the parasitological and/or serological study of their new child to investigate infection by *T. cruzi*.

Seventy newborns of the index cases were studied by PCR and/or by conventional serology (IFI and ELISA IgG) in umbilical cord at birth and venous blood in follow-up, according to established protocols [7]. Congenital infection was confirmed in six newborns (8.6%). In four cases, infection was confirmed by serial PCR and conventional serology in peripheral blood performed between 1 and 12 months of life. Two of the cases without parasitological study at birth were confirmed at 15 months of age by serological tests.

Fifty-seven of 74 previous children of the index cases with ages ranging from 1½ to 15 years were also studied. In ten of these (17.5%), we confirmed infection by *T. cruzi* serology, and in 42% of them the PCR reaction was positive. Six of the children were from rural sectors and four were urban; there were two pairs of infected twins. These probable congenitally infected maternal siblings were born in the period 2000–2010, after the certification of control of the domiciliary vector in the zone [5]. All the children who could talk said they did not know about triatomines. The questionnaire answered by the mothers concurs with this information; all indicated the absence of *T. infestans* in their houses in this period. The results of the serological and/or parasitological study of the children were given to the mothers, together with an indication of the need to treat the confirmed cases precociously according to current international health norms [8]. So far, only eight of the 16 infected children have been treated, due fundamentally to the fear of the parents because of the adverse effects they have observed in adults of the zone treated for chronic Chagas disease [9]. Although the medical team has informed the mothers about the evolution of the disease and the greater tolerance described for congenital and infant cases in the early chronic stage [10], [11], we have not obtained their approval to give nifurtimox to their children. The cases not considered from this group (17 of 74) correspond mainly to children that have migrated to other cities to study.

The mothers of the index cases (maternal grandmothers of the newborns) were also studied; by serology in 73% and clinical records in 27% of the cases. Of the 70 grandmothers (six deceased), infection by *T. cruzi* was confirmed in 57 (81.4%). Their mean age was 56.5 (range 40–85 years). Although this study did not include the clinical condition of the grandmothers in the chronic stage of Chagas disease, we found that 20% of them had been treated with itraconazole [12] or recently with nifurtimox. The grandmothers indicated that 88.6% remembered having been bitten by triatomines, and 95.7% said there were *T. infestans* in houses where they had lived previously, 97.1% of which were built of mud and straw. In the decades 1960–1990, 84.2% reported having lived with domestic and herd animals around the house, especially goats. It is also interesting to note that 48.5% of the grandmothers indicated that close to their former houses of mud and straw there were stone fences, which are an apt habitat for the native species *Mepraia spinolai*, one of the elements of the animal (wild) cycle of *T. cruzi*. This epidemiological situation may be responsible for the interaction between the wild and domestic cycles. Currently, educational measures and vigilance programs for the vector have caused the rural families of the endemic zone to keep their animals away from their house and yard.

Finally, maternal siblings of the index cases were investigated. Of a total of 220 siblings, we were able to interview 152 (69%), confirming infection in 75 (49.3%) of them (36 women and 39 men). The 31% not interviewed included 7% who did not accept serological testing and 24% who had migrated to other regions in search of better job opportunities, especially mining. It is likely that many of them may not know that they are infected.

In summary, this family study of infection by *T. cruzi* found that out of 419 persons interviewed, 218 were infected (70 index cases, six congenital cases, ten children between the ages of 1.5 and 15 years, 57 mothers of index cases (maternal grandmothers) and 75 maternal siblings of the index cases) (Figure 1). We found an average of 3.1 infected members per nuclear family.

The number of individuals infected by *T. cruzi* found in this family study, added to the lack of adequate notification that exists in a number of Latin American countries, lead us to suggest that the prevalence data for different age groups may be greater than the current estimates because it is possible that they consider only the index cases and not their contacts. Recently, it has been proposed that Chagas disease should be systematically investigated in siblings and relatives of infected mothers (serological investigation) [8]. Previous studies fortify this idea [13].

In 1999, Chile was declared free of vector transmission of *T. cruzi* by *T. infestans* [5]. The epidemiological information and results obtained in this study suggest that the decrease of *T. cruzi* infection in groups of lower age may be the result of vector control in highly endemic areas due to the fact that the majority of the children of the index cases were born after the certification that Chile has interrupted transmission by *T. infestans*. On the contrary, most of the mothers, grandmothers, and siblings were born before that date.

One aspect that reveals the clinical history of the interviewed individuals is the lack of pharmacological treatment of index cases, children, their mothers, and maternal siblings during practically their whole lives. All of these, and the new cases infected by other mechanisms without control, such as congenital infection, require sustainable programs of attention and control as has been suggested [8], [14], [15].
